# Sensory and Physico-Psychological Metaphor Comprehension in Children with ASD: A Preliminary Study on the Outcomes of a Treatment

**DOI:** 10.3390/brainsci7070085

**Published:** 2017-07-17

**Authors:** Sergio Melogno, Maria Antonietta Pinto, Gloria Di Filippo

**Affiliations:** 1 Department of Developmental and Social Psychology, “Sapienza” University of Rome, Via dei Marsi 78, 00185 Rome, Italy; mariantonietta.pinto@uniroma1.it; 2 Faculty of Psychology, “Niccolò Cusano” Telematic University - Rome, Via Don Carlo Gnocchi, 3, 00166 Rome, Italy; gloria.difilippo@unicusano.it

**Keywords:** children with ASD, metaphor comprehension, sensory and physico-psychological, treatment, assessment

## Abstract

Recent research into difficulties in figurative language in children with ASD highlighted that it is possible to devise training interventions to overcome these difficulties by teaching specific strategies. This study describes how children with ASD can improve their capability to explain metaphors with a treatment. Two types of metaphors, in the “X is Y” form, were addressed: sensory and physico-psychological. To face the difficulties posed by these metaphors, the adult taught two strategies: inserting the connective “is like” between “X” and “Y”, which transforms the metaphor into a simile; comparing “X” and “Y” by means of thinking maps. Two tests of metaphor comprehension were used, one based on sensory and the other on physico-psychological metaphors. Sixteen 10 year-old children participated into the study, including an experimental group formed by 8 children with ASD (*n* = 4) which had received the treatment, and a control group (*n* = 4) which had not, and 8 typically-developing (TD) children. At the post-test, the experimental group significantly outperformed the controls in explaining both types of metaphors, but only in the sensory metaphors did their performances reach TD children’s levels. These results illuminate how clinical treatment can positively influence the developmental trajectories of metaphor comprehension.

## 1. Introduction

Autism Spectrum Disorder (ASD, henceforth) is a neurodevelopmental disorder characterized by impairments in interaction and social communication, and restricted, repetitive behaviour and interests [1]. Actually, it refers to an extremely heterogeneous condition whose atypical cognitive profiles are still the object of extensive discussion among researchers (see, on this point, [2,3,4,5]). For instance, linguistic competencies in children with ASD can range from total absence of language to fluent language with some difficulties in pragmatic usages [6,7], typically in figurative language.

Recent research has highlighted that it is relevant [8], and possible [9,10,11,12], to devise training interventions to overcome these difficulties by teaching ad hoc strategies. In addition to being clearly helpful for practical reasons, research on treatment can appropriately address some theoretical questions raised by developmental and clinical research on figurative language in children with ASD. For instance, concerning metaphor, researchers [13] recently wondered whether differences between children with and without ASD decrease with age, and also whether children with ASD can improve their competencies, with or without treatment.

In order to understand the nature of the difficulties posed by metaphors, in at least some subgroups of children with ASD [8,14,15], we must recall the prototypical structure of metaphor, which is “X is Y”. In the example “My daddy is a volcano”, the first term, namely, “X-daddy”, is called the “tenor” [16] and is associated on a purely metaphorical plane to the second term, namely “Y-volcano”, which is called the “vehicle” of the metaphor. As X cannot be Y, neither on logical nor on factual grounds, this association poses a semantic conflict, which, nevertheless, can be solved if we think that X resembles Y, in some way.

In other words, some semantic features of X can be viewed as similar to some semantic features of Y, and a common semantic ground can, therefore, be established. On the other hand, to be able to detect a common ground is only a first step towards a satisfactory solution of the semantic conflict, because commonalities have to be adjusted to the characteristics of the tenor. For instance, in the above example “My daddy is a volcano”, the explosive character of the volcano must be adjusted to the behavioural characteristics of a human being [17], such as hyperactivity or hyperproductivity, and the like, depending on the discourse context. In typical development, children gradually come to elaborate strategies to solve this type of semantic conflict, and distinguish between statements based on strictly logical or factual grounds from statements based on general similarities, such as metaphors [18,19]. Nevertheless, this capability may vary as a function of the semantic nature of different types of metaphors. For example, in the so-called “sensory metaphors” [20], X is associated to Y on the basis of perceptual features, such as colour, shape, movement, function, and the like (e.g., “Hair is spaghetti”), whereas in the so-called “physico-psychological” metaphors, a person (X) is associated to an objet (Y) on the basis of more or less abstract features (like in the above example: “Daddy-volcano”).

Some children with ASD [14,15,21,22,23,24,25,26,27,28,29,30], instead, tend to adhere to literal meanings. When requested to explain their position, they often say that the association between X and Y is just impossible and must be refused. Nevertheless, it is possible to show these children that, between the two extremes of totally true and totally false statements, there exists an acceptable intermediate solution, represented by similarities. In order to detect similarities between the tenor and vehicle, and find an appropriate solution to the semantic conflict, a systematic analysis of the meanings of the tenor of vehicle must be implemented. This type of analysis can be transformed into specific strategies that adults can teach.

For instance, Mashal and Kasirer [9] used thinking maps as a tool to enable a group of 20 children with ASD (age range: 12–15 years) to visualize the semantic relations in metaphors. For instance, for the metaphor “train of thought”, children had to write the two terms “train” and “thought” in two bubbles, and their semantic associations in surrounding bubbles. To make children understand the metaphor, the researchers instructed them to write the appropriate associations between train and thought, which, in this case, could point to the idea of “continuity” or of “connected thoughts”. The children had to exclude irrelevant associations between “train” and other words (for example, “car” or “engines”, etc.) as well as between “thought” and other words (for example, “brain” or “in the head”). In a multiple case-study, Persicke and colleagues [10] used a guided procedure to train three children with ASD (age range: 5–7 years) to compare the terms of the metaphors, which, in this case, were embedded in stories. Step by step, each child was guided towards abstracting the relevant shared semantic features while rejecting the irrelevant ones. Melogno, Pinto, and Orsolini [12] report on training used with a single child with ASD (8–10 years) aimed at enhancing metaphor comprehension. The training consisted in teaching two strategies, implemented by means of three main activities. In the first activity, two strategies were taught. The first, called the “X is like Y heuristic”, consisting of exercises training the child to inhibit a literal interpretation, changing a metaphor into the corresponding simile (“X is like Y”). The second strategy, or comparative strategy, was based on the use of thinking maps to search for semantic similarities between X and Y. In the second activity, the child was asked to use unconventional labels to rename objects, or images, or people in a metaphorical sense (for example, “flour” renamed as “snow”). The third activity, “story-matching”, required the child to find the appropriate metaphorical ending to a story, by choosing between two pre-coded alternatives. It is worth noting that all of the studies that described training procedures to improve metaphor comprehension we reported so far highlighted beneficial effects beyond the differences in the age of the participants.

The present study expanded the type of training described in the abovementioned Melogno et al.’s article [12] and was implemented in a small group formed by an adult and four children. The group modality was preferred to the individual one in that it highlights the stimulating role of different perspectives in explaining metaphors. In other words, each interpretation proposed by children addresses a distinct facet of the same metaphor and stimulates the construction of new meanings. However, in this study we only reported on the outcomes of the treatment, as measured at pre- and post-test, while the qualitative analysis of the adult–children’s interactions can be the object of a further work.

## 2. Materials and Methods

### 2.1. Participants

Sixteen children participated into this study, eight diagnosed with Autism Spectrum Disorder (ASD; age range: 9–10 years old) and eight typically-developing children (TD; age range: 9–10 years old). The children with ASD received their diagnosis in a neuropsychiatry center (University of Rome “Sapienza”, according to DSM IV’s [31] (Autistic disorder) criteria (At the time of the diagnosis, these were the criteria that were in force) by using the Autism Diagnostic Observation Schedule (ADOS) [32,33] and Autism Diagnostic Interview-Revised (ADI-R) [34,35]. Four of these children (conventionally named Child F, L, A, and G) formed the experimental group and the other four (Child 1, 2, 3, and 4), the control group. The assignment of the children to each group was made on a random basis. All were males.

The eight TD children (four males and four females) were recruited in a school attended by children of average SES families. Based on teachers’ statements, none of them had any type of behavioural disorder or learning disability. The eight children with ASD were recruited from a larger group on the basis of the following criteria: age range (9–10); both Full Scale IQ and Verbal IQ ≥ 85 (WISC III (At the time of this study, WISC III was the only version of the Wechsler scales available in Italy.) [36,37]; lexical performances (Peabody Picture Vocabulary Test, PPVT), [38,39] and grammatical performances (Test of Reception of Grammar, TROG 2) [40,41] within norms; a drop in figurative language comprehension greater than 1.5 standard deviation below the mean, as measured by a subtest of a battery (APL Medea; it: Abilità Pragmatiche nel Linguaggio Medea; en: Pragmatic Language Abilities Medea) [42]. This subtest requires the child to listen to metaphors (ex: “Mark is a lion”) and idioms (“He always has his head in the clouds”), and explain the meaning (four items), and indicate the picture that matches the appropriate meaning by choosing among four possibilities (four items).

Table 1 provides descriptive statistics for IQ scores (full scale, and verbal and performance scales), vocabulary (PPVT), grammar (TROG 2), and figurative language (APL). Student’s *t* tests for independent groups were applied to all the measures just described, for both the experimental and control groups. The two groups did not differ in age (*t*: –0.8703; df: 6; *p*: 0.4175), Full Scale IQ (*t:* 0.4155; df: 6; *p*: 0.6922), Verbal IQ (*t:* 0.4285; df: 6; *p*: 0.6831), Performance IQ (*t:* 1.3727; df: 6; *p*: 0.2189), vocabulary (*t:* 0.4931; df: 6; *p*: 0.6394), grammar (*t:* –0.3624; df: 6; *p*: 0.7294), and figurative language comprehension (*t:* –0.4902; df: 6; *p*: 0.6413).

### 2.2. The Research Design

After approval from the Ethics Committee of the Department of Pediatrics and Child Neuropsychiatry, “Sapienza University of Rome, we asked for informed consent from the children’s parents, who accepted to having their children involved in this study.

The study applied a before-after design, with an experimental group opposed to a control group, both assessed at pre- and post-test. At time 1 (pre-test), sensory and physico-psychological metaphors comprehension was assessed. At time 2, the treatment was implemented only with the experimental group, and at time 3 (post-test), the same abilities were assessed again in both groups. The study was conducted from June to July.

### 2.3. Measures

To assess metaphor comprehension, we chose two tests measuring the capability to explain the meaning of metaphors, one devised for young children (4–6 years), the Junior Metaphor Comprehension Test (henceforth, Jnr MCT) [43], and the other, the Metaphor Comprehension Test for older children (9–14 years; henceforth, MCT) (A psychologist trained to administer the jnr MCT and the MCT was responsible for the administration and blind to all the issues concerning the intervention.) [44]. Both tests have been validated on TD Italian-speaking children. The Jnr MCT is composed of 12 metaphors embedded in sentences, and 13 metaphors contextualized in four stories. Nearly all of these metaphors are “sensory”, e.g., “The moon is a light bulb”, where “moon” pertains to the domain of “celestial bodies” and “light bulb” to that of “electric devices”. The MCT is composed of 12 physico-psychological metaphorical sentences, such as “The guardian of the prison is a rock”, where “guardian” pertains to the domain of “human beings” and “rock” to the physical domain. In both tests, questions and answers are purely verbal; the administration is preceded by an example the examiner considers together with the child in order to make him/her understand that the items can be analysed on explicit and consistent grounds. No further feedback is given to the child’s answers.

In both tests, the coding system is based on qualitative levels, transformed into quantitative scales, a three-step scale for the Jnr MCT, and a four-step scale for the MCT. Each qualitative level represents a step in the process of solving the semantic conflict inherent in metaphors.

#### 2.3.1. Jnr MCT

- A score of 0 is assigned when the child declares he/she just does not know (elusion of the conflict), or refuses the possibility of using words metaphorically (refusal of the conflict), or interprets the metaphor literally (literal interpretation).
- A score of 1 is assigned when one semantic feature common to both terms of the metaphor is identified. This represents a relevant common ground, based on functional or perceptual characteristics, which partially solves the conflict.
- A score of 2 is assigned when the explanation considers both differences and resemblances between the two terms of the metaphor. This represents an elaborated common ground, which solves the conflict in a more accomplished way.

The maximum score for the Jnr MCT is 50.

#### 2.3.2. MCT

- As in the Jnr MCT, a score of 0 is assigned when the child declares he/she just does not know (elusion of the conflict), or refuses the possibility of using words metaphorically (refusal of the conflict), or interprets the metaphor literally (literal interpretation).
- A score of 1 is assigned when the child provides a common ground between tenor and vehicle based on physical features.
- A score of 2 is assigned when the child provides a more elaborated common ground between tenor and vehicle, where the psychological features are explicitly indicated.
- A score of 3 is assigned when the psychological features that justify the common ground between tenor and vehicle are further refined.

The maximum score for the MCT is 36.

Jnr MCT has high reliability, as measured by Cronbach’s alpha (860), high test-retest correlations (r–tt: 0.848), and high inter-rater agreement, as measured by Cohen’s K (68 for the four year-olds; 0.73 for the five year olds, and 0.74 for the six year olds). 

MCT has high reliability, as measured by Cronbach’s alpha (70), high test-retest correlations: r–tt: 0.83, and high inter-rater agreement, as measured by Cohen’s K (75 for the 9–10 year-olds; 0.74 for the 11 year-olds; 0.67 for the 12 year-olds; 0.81 for the 13 year-olds).

### 2.4. Treatment

The experimental training was based on the theoretical grounds and hypotheses put forward in the abovementioned study by Melogno and colleagues [12]. On the assumption that metaphors are based on a semantic conflict, they propose to approach this type of conflict as a problem-solving activity. To this end, they actively analyze the meanings of both tenor and vehicle in search for a semantic common ground. They also hypothesize that children with ASD can be taught to inhibit literal interpretations, switch flexibly from one semantic feature to another when analyzing the meanings of tenor and vehicle.

Differently from Melogno et al.’s study [12], only the first two activities were implemented. The first activity aimed at teaching strategy 1 (“X is like Y heuristic”) by means of cards (see Table 2). The objective was to induce children to rephrase the expression “X *is* Y” as “X is like Y”. The first activity also included strategy 2 or comparative strategy (thinking maps were used as a tool to search for semantic similarities between X and Y). Both strategies were modelled by the adult, then practised in joint activity, and then the children had to apply them on their own. At the end of each phase of these activities, the adult stimulated a recapitulation of what had been said in the form of a metalinguistic reflection. Table 2 illustrates strategy 1 and 2 in these three phases.

The second activity, called “renaming” had two variants, one with objects and the other with persons. In the first variant (See Table 3, Column a), the adult presented an object or an image and asks the children to rename it (a). Then he is asked to justify the reasons for choosing the new label (b), and to select an image that matches the label chosen for the renaming (c). In the end, he asked to phrase the association between the object (X) and the new label that had been chosen (Y) for renaming in the form “X is Y” (d). In the second variant (see Table 3, Column b), the same pattern applied, except for the selection of the image (point c, above). Renaming addressed three categories: animals, fruit, and objects.

The treatment has been implemented for three weeks, twice a week (six sessions in all), for about 90 min per session. Table 4 shows the schedule of the activities in each session.

In the whole, children have been involved in 12 “X is like Y” exercises, 12 objects, and three person renaming activities. Twelve metaphors have been used, six sensory and six physico-psychological. None of the metaphors used for the assessment was included in the exercises.

## 3. Results

Raw and *t* scores obtained by the experimental group with the Jnr MCT and the MCT, at pre- and post-test, have been compared to those obtained by the control group (Table 5).

To compare the performances of the experimental and control groups with Jnr MCT and MCT, at both pre- and post-test, two Student’s *t* tests have been applied to the standardized total scores obtained by each group. 

Table 6 shows that there were no differences at pre-test, whereas, at post-test, the experimental group performed significantly higher at both Jnr MCT (*t*: 6, 9413; *p* < 0, 0004) and MCT (*t*: 5, 4369; *p* < 0, 0016). It is to be noted that, at the MCT (post-test), only one child (Child L) reached a T score within the average range (40–60) while the others were positioned at lower-average level (30–40).

As the upper age for the Jnr MCT is six years, whereas all our participants were between nine and 10 years, the performances of the experimental children were compared to those of eight TD children of the same age. In this case, as the standardized scores only apply up to six years, we performed the Student’s *t* test on the raw scores (Exp: m.: 39, 75; sd: 4, 4253; TD: m.: 43,25; sd: 2, 3145). At post-test, children with ASD performed very similarly to the TD children, and the difference was only close to significance (*t*: –1, 8422; *p* < 0, 09552).

## 4. Discussion

In this article, we presented a preliminary study on training whose aim was to enhance the comprehension of sensory and physico-psychological metaphors in children with ASD. The distinction between these two types of metaphors is based on developmental literature, where it appears that explaining metaphors in childhood may vary as a function of the semantic complexity of these metaphors, with an advantage for the sensory type [20,28]. 

Eight children with ASD, internally homogeneous for age (age range: 9–10 years): intellectual level, basic lexical and grammatical competencies, and a weakness in figurative language comprehension, participated into this study. An experimental group and a control group were created on a random basis, the experimental group being chosen in view of the treatment. Both groups were assessed in metaphor comprehension before and after the treatment (pre- and post-test) by means of two metaphor comprehension tests, the Jnr MCT [43], which measures sensory metaphors, and the MCT [44], which measures physico-psychological metaphors.

The training consisted of three main activities led by an adult together with the children. These activities were: (a) the “X is like Y heuristic”, where children were taught to insert the connective “like” between the two terms of a metaphor in order to convert it into a simile; (b) the “comparative strategy”, using thinking maps to stimulate comparisons between the features of each term of the metaphor; and (c) renaming objects and individuals with metaphorical labels [12].

After six sessions, the experimental children significantly improved their capability to explain both types of metaphors, as measured by each test of metaphor comprehension. On the other hand, there were no significant improvements in the control group when comparing their performances at pre- and post-test. It might be argued that the repetition of the same tests after a relatively short time might have facilitated the retrieval of correct answers. However, in both metaphor comprehension tests, the request is not to identify a pre-coded answer but to explain the meaning of the metaphors in one’s own words. In addition, as pointed out in the Materials and Methods section, children received no feedback on their answers during the administration so that they could not memorize a particular solution. Lastly, if the improvements of the experimental group at post-test were due to the repetition of the tests, we could not explain why the control group did not improve at all, although it received exactly the same tests.

The experimental group was also compared to a TD group, matched by age, in order to see whether the advance in sensory metaphors, which could be attributed to the training, raised them to the level shown by 10 year-old TD children. At post-test, the experimental children’s performances at the Jnr MCT were just slightly lower than those of the TD children. It is to be noted that, after the training, the performances at the MCT were still at a lower-average level for three out of four children. This outcome can be explained by the greater semantic complexity of the metaphors of the MCT, as these require two distinct domains to be bridged, one outer and tangible, and the other, inner and immaterial, as in the above “daddy-volcano” example. In other words, to interpret a physico-psychological metaphor, it is not sufficient to identify the common features between tenor and vehicle, it is also necessary to adjust them to the characteristics of the tenor. Some characteristics must be inhibited as irrelevant, in order to shift from one of the meanings of a word to other possible meanings [45,46]. In addition, physico-psychological metaphors require to handle psychological knowledge and mental lexicon, which, for children with ASD, represent a typical weakness, and this might account for their generally fuzzy and incomplete explanations [47].

On qualitative grounds, it is worth noting that, in our study, children sometimes showed an interesting evolution in the course of the same activity—be it metaphor explanation or renaming—from literal answers to more flexible and appropriate interpretations. For instance, during a joint activity, Child G and Child F (Table 2, turn 2 and 3) initially refused the metaphor very firmly (“But it’s absolutely fake!”; “It’s impossible!”). In a later turn (turn 12), Child F started analyzing some relevant features of the metaphor at hand. Child G synthesized elements coming from Child F’s analysis (turn 15) and his own comment (turn 9): “He is sitting next to you, so he’s sticky just as jam sticks to bread”. In turn 18, Child G’s interpretation became fully psychological: “He’s kind because he lends his things. If you forget your eraser, he will lend it to you and you must thank him. He is your best friend.” 

Likewise, in object renaming, Child F passes from a tautological renaming (turn 2) to a spontaneous justification of other children’s renamings (turn 12 and 16), up to his own, appropriate, new label. A future study could highlight how this type of evolution reflects the ways in which the adult stimulates children to go beyond their first reactions, by repeating and recapitulating their answers and by asking them to justify their positions. 

In this study, we can point to two essential novelties. The first one is that the treatment addressed two types of metaphors, sensory and physico-psychological. This was a double challenge in that these types represent two different levels of semantic complexity and, more importantly, this distinction is hardly considered in the literature on children with ASD. The other novelty is the group modality, which seems to amplify the interactions between the participants, and, as a consequence, the possibilities to identify a common ground between tenor and vehicle. Although the analysis of these interactions was not the primary focus of this paper, excerpts in Table 2 and Table 3 provide several examples of how the participants handled relevant meanings. An in-depth analysis of these aspects of the treatment can be the object of a future work. 

We would also like to point to two limitations. Firstly, the sample size is clearly restricted, which gives this study a preliminary character; secondly, we only considered the “X is Y” form of metaphors, which is prototypical, but not exhaustive. Therefore, the generalizability of the outcomes of the treatment should be verified also with other types of metaphors and metaphorical usages, such as metaphorical adjectives and verbs. This would enable us to verify whether the training can produce a “near transfer effect,” that is, enhancing the capability of handling metaphorical usages, in general; thirdly, there has been no follow-up study to test the solidity of the results across time. As the research was conducted during summer, we could not also check for the transfer of the results to academic competencies, for example on text comprehension. 

We believe that the results, although preliminary, can contribute to the theoretical debate on metaphor comprehension in children with ASD. One of the gaps in the literature in this field is that the samples considered are generally very heterogeneous. In our study, instead, the sample was considerably homogeneous in terms of age range, IQ, and basic semantic and grammatical competencies, which were all adequate. The apparent adequacy of the profile of this subgroup reopens the debate on the type of cognitive and linguistic skills that are necessary to understand figurative language, or on the integration of these skills [9,14,25,28,30]. On the other hand, the outcomes of our study suggest that a clinical treatment can at least partially contribute to the evolution of metaphor comprehension in these children with ASD.

Future research should explore the relevant changes that take place in metaphor comprehension from early childhood to adult age in individuals with ASD. Among the many factors involved in this evolution, specific treatments represent a significant concurrent element.

## Figures and Tables

**Table 1:** Descriptive statistics for IQ scores (Full scale, Verbal, Performance). Peabody, TROG 2, APL., in the experimental and control groups (mean, standard deviation, range).

	Age			Full Scale IQ			VIQ			PIQ			Peabody			TROG 2			Metaphors APL		
	Mean	SD	Range	Mean	SD	Range	Mean	SD	Range	Mean	SD	Range	Mean	SD	Range	Mean	SD	Range	Mean	SD	Range
Exp. G.	121.75	2.2173	119–124	99.25	3.8622	95–103	90.75	2.0615	89–93	108.25	4.5734	103–113	103.00	5.3541	98–10 9	101.00	5.2281	97–108	–1.8425	0.1286	–1.65–1.92
Contr. G.	123.00	1.8257	121–125	98.25	2.8722	95–102	90.00	2.8284	86–92	102.00	7.8740	95–113	101.50	2.8867	98–105	102.25	4.5000	97–108	–1.9025	0.2082	–1.65–2.16

**Table 2:** Examples of activities with strategy 1 (“X is like Y heuristic”), and 2 (Comparative strategy).

**Example of modelling**
Adult: “When you listen at someone saying ‘a daisy is an umbrella’, you certainly know that a daisy *cannot* be an umbrella. A daisy is a flower and an umbrella is an object. To understand the meaning of this sentence, I will use my first strategy. I will put “Is like” between “daisy” (X) and “umbrella” (Y) and the sentence becomes “A daisy is like an umbrella”.
**Strategy 1: “Is like strategy”**
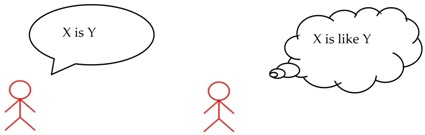
Now, I will use my second strategy. I’ll be thinking of the characteristics of the daisy and write them down here in my thinking map. Then, I’ll write that daisies are flowers, that they have a stem, a corolla, they are white….etc.
Now, it’s umbrella’s turn. I’ll be writing its characteristics down in my map. An umbrella has a dome, a handle, a tube, ribs, and it protects us from the rain. Right. Now, I’ll see which characteristics are appropriate for both daisies and umbrellas. For instance, “Are they both flowers? No. Then I’ll reject ‘flowers’. “Daisies have a stem”. Umbrellas don’t have stems, they have tubes. Still, we can say that stems look like tubes. And also, the open dome of an umbrella looks like the corolla of a daisy….Then, I got it: a daisy has a shape that looks like an open umbrella.”
**Strategy 2: Comparative strategy**
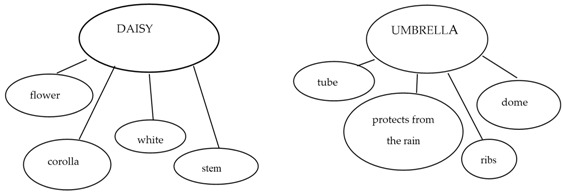
**Metalinguistic reflection**
Then, we may state that the guy who said “A daisy is an umbrella” actually meant that these objects share the same shape.”
**Example of joint activity**
(1) Adult: “What does it mean that ‘My classmate is a butter and jam sandwich’?” (2) Child G: “But it’s absolutely fake! A classmate is a living being. Butter and jam are food for breakfast”. (3) Child F: “It’s impossible. It’s a grammatical error. The right sentence is: ‘My classmate has a butter and jam sandwich’”. (4) Adult: “Ok, ok. Put this way, the sentence looks like an error. Let’s try to use our first strategy…….” (5) Child L: “let’s put ‘is like’ between “classmate” and “butter and jam”, and then the sentence becomes ‘My classmate is like a butter and jam sandwich’”. (6) Adult: “That’s it: ‘My classmate is like a butter and jam sandwich’”. Now, let’s use our second strategy (Comparative strategy). (7) Adult: “In this bubble I’ll write ‘classmate’. Which characteristics can you tell me about ‘classmate’?”. (8) Child L: “Living being”. (9) Child G: “He is sitting next to you” (10) Child L: “He has a backpack like you”. (11) Adult: “And now, tell me about the characteristics of ‘butter and jam sandwich’”. (12) Child F: “‘food’, ‘sticky’, ‘sweet’”. (13) Child A: “‘flour’, ‘sugar”. (14) Adult: “Ok. Now, let’s see which characteristics we’ll keep and which ones we’ll reject.” (Children reject all the characteristics except two: ‘sticky’ and sweet’). (15) Child G: “He is sitting next to you, so he’s is sticky just as jam sticks to bread”. (16) Adult: “Very good! Now, we only need to find something for ‘sweet’. When we say ‘this classmate is ‘sweet’, what do we mean?” (17) Child A: “That he eats bread and jam.” (18) Child G: “That he is kind because he lends his things. If you forget your eraser, he will lend it to you and you must thank him. He is your best friend.”
**Metalinguistic reflection**
The adult recapitulates and proposes the following solution: “When one says ‘my classmate is like a butter and jam sandwich’ it actually means this classmate is a close friend.”
**Example of autonomous activity**
Children are given a written sentence: “In summer, meadows are blankets”. Then, they individually use strategy 1 and 2, and at the end, the adult asks each of them: “What does it mean, then, that ‘In summer, meadows are blankets’?”.
**Metalinguistic reflection**
The adult makes the children notice that various answers are acceptable, as, for instance: ”Meadows can be used like blankets because they cover the earth with a soft surface, and you can play on it in various ways.”

**Table 3:** Examples of renaming activities.

(a) Object Renaming	(b) Person Renaming
In a circle time, the adult shows a tennis ball and says:	In a circle time, the adult proposes the renaming game in the following terms:
(1) “Let’s play our renaming game. We will look at this and try to give it a new name. (2) Child F: “A small ball”. (3) Child G: “Tennis”. (4) Adult: “Ok. But now let’s try to invent a new name for this object“. (5) Child L: “A lemon”. (6) Adult: “Then, a tennis ball is a lemon. Why?” (7) Child L: “It’s yellow”. (8) Adult: “Oh, then it’s for the color?” (9) Child G: “A yellow stone”. (10) Adult: “Why?” (11) Child G: “Because they both roll”. (12) Child F: “Because it’s a bit hairy”. (13) Child A: “Then, it’s the sun….The sun is a planet”. (14) Child F: “No! The sun is a star!” (15) Child A: “It’s a planet because of the shape!” (16) Child F: “No, it’s because of the color”. (17) Adult (Addressing child F): “You said the tennis ball is a bit hairy. How could we rename it?” (18) Child F: “A ball of cotton.” At this point, the adult asks children to match the images related to the new labels to the appropriate object. 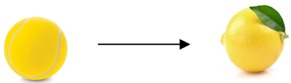 Lastly, he invites children to phrase the outcome of the renaming in the form “X is Y”. Ex: “A tennis ball is a lemon”.	(1) Adult: “Let’s pretend a person can be renamed as an animal. (Addressing child F). If you were an animal, what would be your name?” (2) Child F: ……“A lion”. (3) Adult: “Lets’ ask child F why he renamed himself as a lion”. (4) Child F: “Because a lion is strong”. (5) Child L: “A lion is a carnivorous mammal”. (6) Child G: “It’s a feline”….but the lion is different from the lioness because it has a mane”. (7) Adult: “Then, a lion is strong, carnivorous, a feline and has a mane. Then, if we say ‘he is a lion’ we are not talking about an animal but a person. And what does it mean that ‘he is a lion’?” (8) Child G: “He has much hair and a beard like a mane”. (9) Adult: “Ok, let’s go on. (Addressing child L) And you, if you were an animal?”. (10) Child L: “Oh, I got it. Sure, no doubt, I would be a horse.” (11) Adult: “Try to explain us”. (12) Child L: “When it’s small, it’s a foal. Then it grows up and becomes a horse. I adore horses.” (13) Adult: “But when we say someone is a horse, what do we mean?” (14) Child A: “That he can practise many sports and games”. (15) Adult: “Can you explain it better?” (16) Child A: “A horse can swim, run and jump. Then, it’s a sportsman.”

**Table 4:** Schedule of the activities in each session.

	1st	2nd	3rd	4th	5th	6th
**“X is like heuristics” and comparative strategy**	Two activities modeled by the adult with sensory metaphors	Two joint activities with sensory metaphors	One joint activity with a sensory metaphor and one modeled activity with a physico-psychological metaphor	Two joint activities with two physico-psychological metaphors	Two joint activities with two physico-psychological metaphors	Two autonomous activities, one with a sensory metaphor, and the other with a physico-psychological metaphor
**Object renaming**	Four objects	Four objects	Four objects			
**Person renaming**				Animals	Fruit	Objects

**Table 5:** Raw and *t* scores at the Jnr MCT and MCT, pre- and post-test. Experimental and control groups.

	Jnr MCT Pre-test		Jnr MCT Post-Test		MCT Pre-test		MCT Post-Test	
	Raw Score	*t* Score	Raw Score	*t* Score	Raw Score	*t* Score	Raw Score	*t* Score
**Exp Child L**	27	60	43	89	3	29	12	49
**Exp Child A**	20	47	35	74	2	27	8	40
**Exp Child G**	20	47	44	91	3	29	8	40
**Exp Child F**	14	36	37	78	2	27	7	38
**Con Child 1**	16	40	17	41	1	25	2	27
**Con Child 2**	21	49	22	51	2	27	2	27
**Con Child 3**	24	54	24	54	3	29	3	29
**Con Child 4**	20	47	20	47	2	27	3	29

**Table 6:** Student’s *t* tests for independent groups (experimental versus control) applied to Jnr MCT and MCT.

	Jnr. MCT								MCT							
	Pre-Test				Post-Test				Pre-Test				Post-Test			
	Mean	*t*	*p*	DS	Mean	*t*	*P*	DS	Mean	*t*	*p*	DS	Mean	*t*	*p*	DS
Exp. G	47.50	0.00	1.00	9.8149	83.00	6.9413	0.0004	8.2865	28.00	1	0.3559	1.1547	41.75	5.4369	0.0016	4.9244
Contr. G	47.50			5.8022	48.25			5.6199	27.00			1.6329	28.00			1.1547
